# Polyphyllin II inhibits thyroid cancer cell growth by simultaneously inhibiting glycolysis and oxidative phosphorylation

**DOI:** 10.1515/med-2025-1286

**Published:** 2025-10-27

**Authors:** Jianwei Sun, Ding Ding, Qian Xiang, Mengyang Zheng, Mingming Dai

**Affiliations:** Department of Ultrasound, Fifth Affiliated Hospital of Kunming Medical University, 17 South Goldenlake Rd., Gejiu, Yunnan, 661000, China; Department of Endocrinology, Fifth Affiliated Hospital of Kunming Medical University, Gejiu, Yunnan, 661000, China

**Keywords:** polyphyllin, glycolysis, oxidative phosphorylation, proliferation, invasion, migration, apoptosis

## Abstract

**Background:**

Thyroid cancer is the most common malignancy of the endocrine system, and effective treatments for metastatic disease are still lacking. Targeting both glycolysis and oxidative phosphorylation (OXPHOS) simultaneously represents a novel approach to cancer therapy. While polyphyllin has been shown to modulate cellular metabolism in various cancers, its role in thyroid cancer remains unexplored.

**Purpose:**

This study aimed to explore the antitumor effects and underlying mechanisms of polyphyllin in thyroid cancer.

**Methods:**

Thyroid cancer cells were treated with varying concentrations of Polyphyllin I, II, VI, and VII. Cell viability was assessed using the CCK-8 assay to identify the most effective polyphyllin compound and its optimal dosage. Colony formation and EdU incorporation assay were performed to evaluate cell proliferation, while Transwell assays were used to assess cell invasion. Cell migration ability was examined using the wound healing assay. The effect of Polyphyllin II on OXPHOS was evaluated using an extracellular oxygen consumption rate (OCR) assay kit. Glucose uptake, lactate production, glycolysis-related protein expression, and the extracellular acidification rate (ECAR) were measured to assess the effects of Polyphyllin II on glycolysis in thyroid cancer cells. Flow cytometry and western blotting were conducted to detect apoptosis.

**Results:**

Polyphyllin I, II, VI, and VII all inhibit the proliferation of thyroid cancer cells, with Polyphyllin II showing the most potent inhibitory effect. Polyphyllin II suppresses cell proliferation, invasion, and migration of thyroid cancer cells, while also promoting apoptosis. Mechanism studies reveal that Polyphyllin II inhibits extracellular OCR, basal respiration, maximum respiration, ATP-linked respiration, spare respiration capacity, glucose uptake, lactate production, glycolytic rate-limiting enzymes, and the ECAR in thyroid cancer cells.

**Conclusion:**

Polyphyllin II simultaneously inhibits glycolysis and OXPHOS, thereby suppressing the invasion, migration, and proliferation of thyroid cancer cells, while also promoting apoptosis.

## 1 Introduction

Thyroid cancer is the most common malignant tumor of the endocrine system, with both incidence and mortality rates increasing annually [1]. While localized diseases can usually be treated with surgery, patients with metastatic diseases have limited treatment options, poor overall survival, and a lack of effective strategies [2,3]. Therefore, there is an urgent need to develop new treatment methods for metastatic thyroid cancer.

Oxidative phosphorylation (OXPHOS) and glycolysis are the two major pathways for ATP production [4]. A key metabolic characteristic of tumor cells is the Warburg effect, particularly aerobic glycolysis, which allows cancer cells to survive and proliferate rapidly in the malnourished tumor microenvironment [5,6]. Glycolysis provides suitable targets for the development of anti-tumor drugs and offers hope for tumor treatment. However, inhibiting glycolysis solely through the use of potential pharmacological inhibitors can only inhibit growth, but it is not enough to completely eradicate cancer [7]. This limitation arises from the significant metabolic heterogeneity of tumor cells, which can adapt their metabolism based on environmental conditions. When glycolysis is inhibited, cancer cells can reprogram their energy metabolism and shifting toward glutamine metabolism to fuel the tricarboxylic acid cycle, ultimately producing ATP via OXPHOS to sustain survival [7–9]. In addition, studies have shown that both glycolysis and OXPHOS processes can occur simultaneously during the evolution of the same malignant tumor [10]. Therefore, developing drugs that simultaneously target both glycolysis and OXPHOS may offer a novel strategy to drive tumor cells toward death. According to literature reports, inhibiting either OXPHOS or glycolysis can suppress the growth of thyroid cancer [11,12]. However, drugs that simultaneously inhibit both OXPHOS and glycolysis in thyroid cancer have not been reported yet.

In recent years, active ingredients extracted from traditional Chinese medicine have been widely reported for their potential in treating thyroid cancer. For example, Hu et al. [13] reported that Alantolactone, a terpenoid extracted from the traditional Chinese medicinal herb *Inula helenium* L., induces both apoptosis and GSDME-dependent pyroptosis in anaplastic thyroid cancer through a reactive oxygen species (ROS)-mitochondria-dependent caspase pathway. *Rhizoma Paridis*, a traditional Chinese medicine, contains steroidal saponins as its most effective components [14]. Various saponins from Rhizoma Paris – such as total saponins, Polyphyllin I, Polyphyllin II, Polyphyllin VI, and Polyphyllin VII – have demonstrated strong antitumor activities in different cancers, including breast cancer, lung cancer, colorectal cancer, hepatocellular carcinoma, and gastric cancer [15]. Although Polyphyllin has been demonstrated to induce cell death in various cancers, its impact on cancer cell metabolism remains poorly understood and warrants further investigation. Therefore, this study aimed to explore the anticancer effects and underlying mechanisms of Polyphyllin, a bioactive compound derived from *Rhizoma Paridis*, in thyroid cancer.

## 2 Materials and methods

### 2.1 Cell culture

Human thyroid cancer cells (FTC133 and TPC-1) and normal human thyroid cells Nthy-ori 3-1 were purchased from the Chinese Academy of Science Cell Bank. All cell lines were cultured in RPMI-1640 medium (Gibco, USA, cat: 11875119) supplemented with 10% FBS (Gibco, USA, cat: 10099141C) and 1% ampicillin/streptomycin (Gibco, USA, cat: 15140112), and cultured at 5% CO_2_ with 37°C (Thermo Fisher, USA).

### 2.2 CCK-8

Cell proliferation was assessed using a CCK-8 assay kit (Dojindo, Japan, cat: CK12). Nthy-ori 3-1, FTC133, and TPC-1 cells were seeded in 96-well plates (Corning, USA, cat: 7007) and treated with varying concentrations of Polyphyllin I, II, Ⅲ, VI, and VII. After 24 h of treatment, 10 μL of CCK-8 reagent was added to each well. After 1 h of incubation, the absorbance was measured at 450 nm and IC_50_ values were calculated.

### 2.3 Colony formation assay

FTC133 and TPC-1 cells were seeded into six-well plates and cultured for 24 h to allow for cell adhesion. Following adhesion, the cells were treated with Polyphyllin II for 24 h. After treatment, the cells were maintained in culture for 7 days to facilitate colony formation. Once colonies had formed, they were fixed with 4% paraformaldehyde (Beyotime, China, cat: P0099-100mL) for 30 min, and then stained with 0.1% crystal violet (Yeasen, China, cat: 548-62-9) for 30 min. The plates were imaged, and colony quantification was performed using ImageJ software. All experiments were conducted in triplicate.

### 2.4 5-Ethynyl-2′-deoxyuridine (EdU) incorporation assay

Cells were cultured in six-well plates, and a 2× EdU working solution (20 μM) was prepared according to the manufacturer’s instructions (Beyotime, China, cat: C0071S). An equal volume of pre-warmed (37°C) 2× EdU working solution was added to the wells to achieve a final concentration of 1×, and the cells were incubated for an additional 2 h. After EdU labeling, the cells were fixed for 30 min and then permeabilized for another 30 min. The Click Additive Solution was prepared and 0.5 mL of Click reaction solution was added to each well, followed by a 30 min incubation. Afterward, nuclear staining was performed, and the samples were observed under a fluorescence microscope. The maximum excitation wavelength of Azide 488 is 495 nm, and the maximum emission wavelength is 519 nm.

### 2.5 Transwell

FTC133 and TPC-1 cells (1 × 10^5^ cells/well) were seeded into the upper chamber of a Transwell plate (Beyotime, China, cat: FTW064-12Ins) supplemented with Matrigel matrix (Beyotime, China, cat: C0372-1mL), and medium was added to the lower chamber. The cells were incubated at 5% CO_2_ and 37°C for 24 h. After incubation, the pore plate was removed, and the cells that did not penetrate the membrane were wiped away. The membrane was then fixed with 95% ethanol solution (Aladdin, China, cat: A112717) for 15 min. The cells were stained with 0.1% crystal violet (Beyotime, China, cat: C0121-100mL) for 10 min, washed three times with PBS solution (Procell system, China, cat: PB180327), and air-dried. Six random regions were selected for imaging under an inverted microscope (Nikon, Japan). Cell counting was performed using ImageJ software (USA, version 1.8.0).

### 2.6 Wound healing

Cells were cultured in a 12-well plate and incubated for 24 h. The culture medium containing Polyphyllin II was then replaced with serum-free medium. Using a 200 μL pipette tip, a straight line was drawn on the cell monolayer to create a “wound”. At 0 and 24 h after the experiment, the healing of the scratch area was observed and recorded under a microscope. The distance of cell migration or the healing area at different time points was quantified using ImageJ software to analyze the cell migration rate.

### 2.7 Extracellular oxygen consumption rate (OCR)

Extracellular OCR of thyroid cells was measured using the Extracellular OCR Plate Assay Kit (Dojindo, Japan, cat: E297). Oligomycin (1 μM, Dojindo, Japan, cat: E297) was added to measure OCR associated with ATP production. Carbonyl cyanide-4-trifluoromethoxyphenylhydrazone [FCCP] (2 μM, Dojindo, Japan, cat: E297) was added to determine maximum respiration. Non-mitochondrial respiration was assessed by adding rotenone (0.5 μM, Dojindo, Japan, cat: E297) and antimycin A (0.5 μM, Dojindo, Japan, cat: E297). The following calculations were used: Basal respiration = overall OCR − non-mitochondrial respiration; ATP production = overall OCR − OCR after oligomycin treatment; Maximum respiration = OCR after FCCP treatment − non-mitochondrial respiration; Spare respiratory capacity = maximum respiration – basal respiration.

### 2.8 Measurement of glucose uptake levels

Collect 10^6^ cells into a centrifuge tube (Corning, USA, cat: 430790) and centrifuge at 8,000*g*, 25°C, for 10 min. Discard the supernatant and add 200 μL of distilled water. Place the sample on ice and lyse the cells using ultrasonic waves (200 W power, 3 s of ultrasonication with 10 s intervals, repeated 30 times). Heat the sample in a 95°C water bath for 10 min, allow it to cool, and centrifuge again at 8,000*g*, 25°C, for 10 min. Collect the supernatant for further use. Preheat the spectrophotometer for at least 30 min, adjust the wavelength to 505 nm, and use distilled water for zeroing. Mix reagent two and reagent three in a 1:1 volume ratio, mix thoroughly, and incubate the mixture in a 37°C water bath for 15 min. Following the instructions of the reagent kit (Abcam, UK, cat: ab136955), react the sample with the mixed reagent, measure the absorbance at 412 nm, and calculate the glucose uptake level of the cells based on the standard curve.

### 2.9 Measurement of lactate production

Prepare the reaction mix according to the instructions of the lactate uptake assay kit (Abcam, UK, cat: ab83429). Collect 2 × 10^6^ cells in a centrifuge tube, wash once with PBS, and add 200 µL of Assay Buffer XII/Lactate Assay Buffer. Pipette up and down several times to homogenize the cells, then centrifuge at top speed at 4°C for 5 min. Collect the supernatant, transfer it to a clean tube, and keep it on ice. Add 50 µL of background reaction mix to the sample, gently mix, and incubate at room temperature for 30 min. Finally, measure the absorbance at 450 nm using a microplate reader.

### 2.10 Western blot

Cells were lysed with RIPA buffer (LMAI; China; cat: LM801001C) containing a protease inhibitor. The lysate was incubated on ice for 30 min and then centrifuged at 14,000 rpm for 15 min. The supernatant was collected, and the protein concentration was measured using a BCA kit (Abcam, UK, cat: ab102536). Proteins were separated by SDS-PAGE (Solarbio; China; cat: P1200) and transferred to PVDF membranes (Millipore, USA, cat: IPFL00010). After blocking with skim milk at room temperature for 1 h, the membrane was incubated with primary antibodies (PCNA, GLUT1, HK2, LDHA, PGK1, GPI, GAPDH, and β-actin, all diluted 1:5,000; Biotechne, China) at 4°C overnight. The membrane was then incubated with a secondary antibody conjugated with horseradish peroxidase at room temperature for 1 h. Signals were detected using ECL chemiluminescent kit (Vazyme, China, cat: E411-04). All images were analyzed using ImageJ software.

### 2.11 Extracellular acidification rate (ECAR)

After preparing the working solution according to the kit instructions (Elabsciene, China, cat: E-BC-F069), collect 1 × 10^6^ cells and resuspend them in 200 μL of Reagent I. Dispense the prepared cell suspension into a microplate by adding 100 μL of cell suspension to each test well. For blank control wells, add 100 μL of Reagent I. Subsequently, incubate the microplate at 37°C in the dark for 30 min, while preheating the fluorescence microplate reader and setting the temperature to 37°C. After incubation, add 100 μL of the prepared working solution to each well. Using a fluorescence microplate reader, measure the fluorescence at an excitation wavelength of 490 nm and an emission wavelength of 535 nm in real time. Record fluorescence values every 5 min for a total duration of 100 min. Finally, plot the fluorescence values against time to generate a curve and calculate the ECAR using data from the linear portion of the curve.

### 2.12 Flow cytometry

After collecting the cells, centrifuge to pellet them and remove the supernatant. Then, resuspend the cells in PBS + FBS buffer. Following the instructions of the apoptosis assay kit (MULTI SCIENCES, China, cat: AP105), add Annexin V-FITC dye and incubate at room temperature for 10 min. Next, add PI dye and incubate at room temperature for another 10 min. Subsequently, analyze the cells using flow cytometry, observing the dual staining of Annexin V-FITC (green fluorescence) and PI (red fluorescence). Finally, perform quantitative analysis of the data using FlowJo software.

### 2.13 Statistical analysis

All results are expressed as mean ± standard deviation. Data analysis and image production were carried out using GraphPad Prism 8. All analyses were performed by *T*-test. *P* < 0.05 indicated statistical significance.

## 3 Results

### 3.1 Polyphyllin inhibits the proliferation of thyroid cancer cells

**Figure 1:**
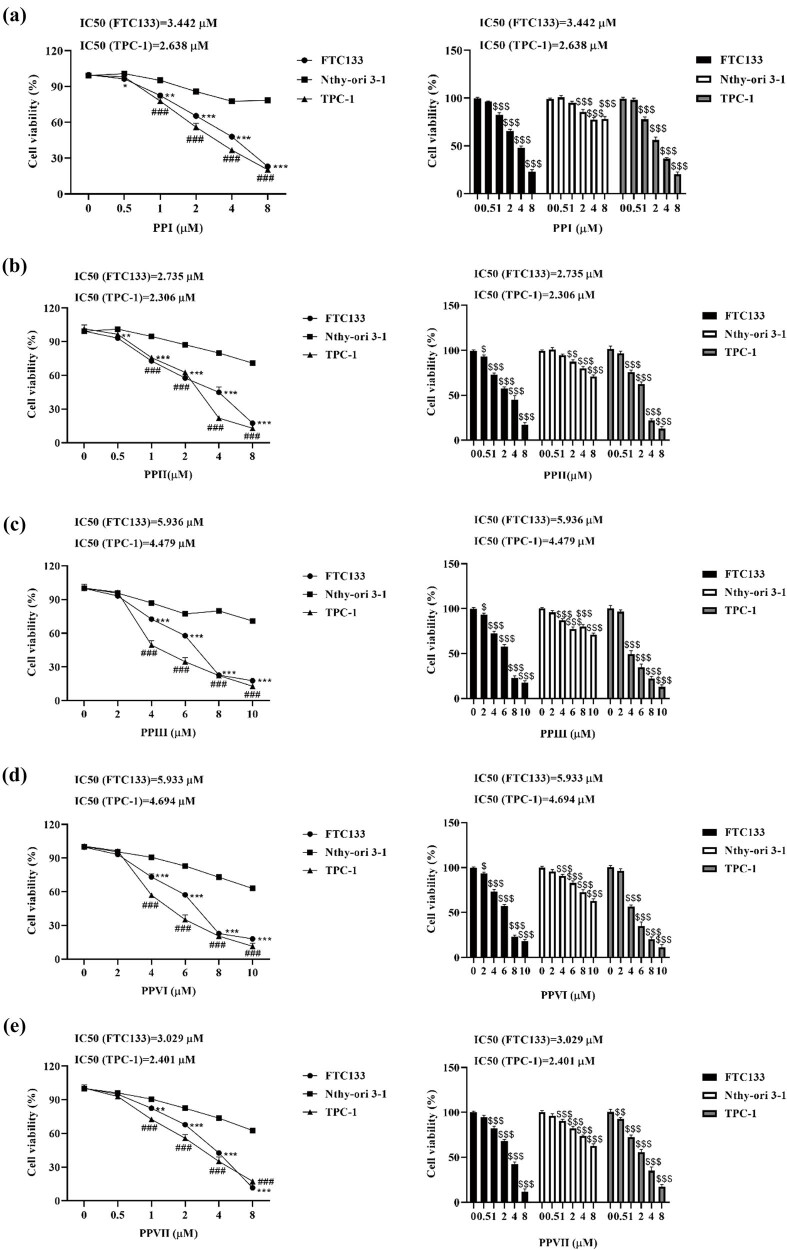

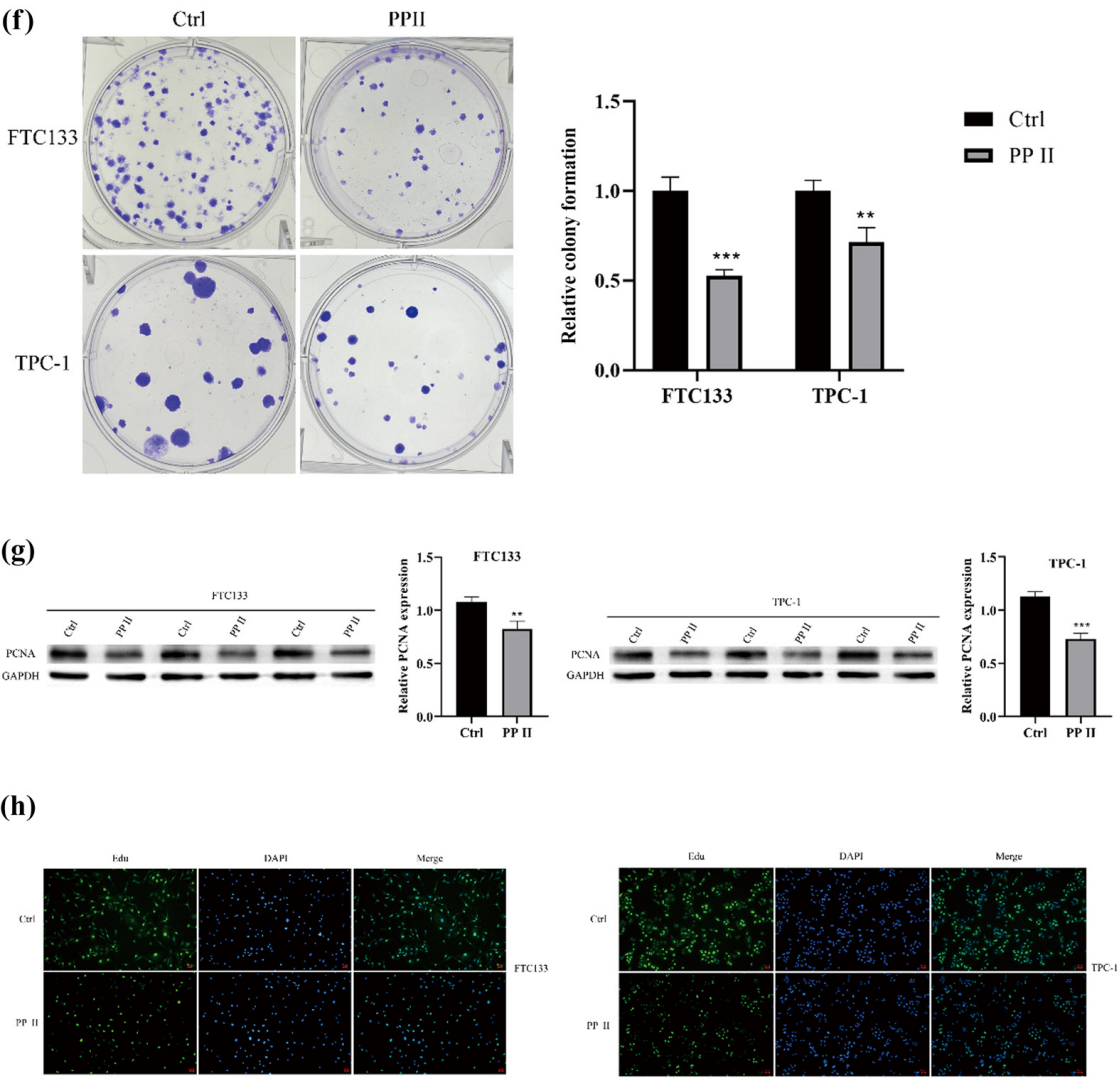
Polyphyllin inhibits the proliferation of thyroid cancer cells. (a)–(e) Nthy-ori 3-1, FTC133, and TPC-1 cells were treated with varying concentrations of polyphyllin I (a), II (b), III (c), VI (d), and VII (e), and cell viability was assessed using the CCK-8 assay. (f) The clonogenic assay was used to evaluate cell proliferative ability. (g) Western blot was used to detect the expression of PCNA. GAPDH was used as an internal control. (h) The incorporation of EdU was used to measure cell proliferation. (a)–(e) FTC133 compared with Nthy-ori 3-1, *p < 0.05, **p < 0.01, ***p < 0.001, TPC-1 compared with Nthy-ori 3-1, ^#^ *p* < 0.05, ^##^ *p* < 0.01, ^###^ *p* < 0.001, compared with 0 μM, ^$^ *p* < 0.05, ^$$^ *p* < 0.01, ^$$$^ *p* < 0.001. (f) and (h) Compared with Ctrl, *p < 0.05, **p < 0.01, ***p < 0.001. PP: Polyphyllin.

After treating Nthy-ori 3-1, FTC133, and TPC-1 with different concentrations of polyphyllin I, II, Ⅲ, VI, and VII, CCK-8 was used to detect cell viability. The results showed that polyphyllin I, II, Ⅲ, VI, and VII inhibited the proliferation of thyroid cancer FTC133 and TPC-1 cells, and high-dose polyphyllin also had certain cytotoxic effects on normal Nthy-ori 3-1 cells (Figure 1). Based on the half-maximal inhibitory concentration (IC_50_), polyphyllin II demonstrated the strongest inhibitory effect (Figure 1b). Moreover, polyphyllin II, at concentrations up to 1 μM, showed no cytotoxicity to Nthy-ori 3-1 cells (Figure 1b). Therefore, 1 μM polyphyllin II was selected for subsequent experiments. After treatment with polyphyllin II, the clonogenic ability (Figure 1f), protein expression of proliferating cell nuclear antigen (PCNA) (Figure 1g), and EdU incorporation (Figure 1h) were significantly downregulated in FTC133 and TPC-1 cells.

### 3.2 Polyphyllin II inhibits the invasion and migration of thyroid cancer cells

**Figure 2:**
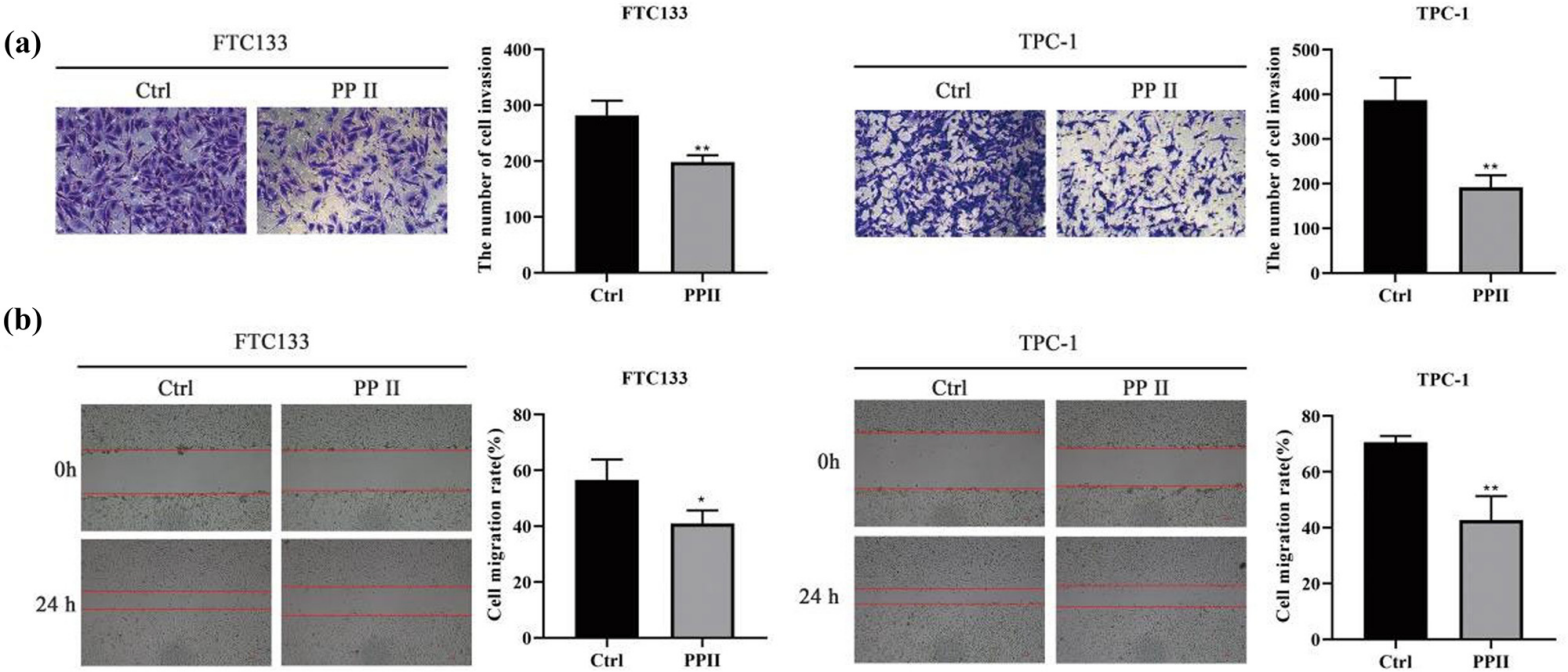
Polyphyllin II inhibits the invasion and migration of thyroid cancer cells. (a) Transwell assay was used to evaluate cell invasion ability. (b) Wound-healing experiments were conducted to assess cell migration ability. Compared with Ctrl, *p < 0.05, **p < 0.01.

FTC133 and TPC-1 cells were treated with 1 μM polyphyllin II. Transwell assays and wound-healing experiments were performed to assess the invasion and migration of both cell lines. The results showed that Polyphyllin II significantly inhibited the invasion and migration of FTC133 and TPC-1 cells, as shown in Figure 2a and b.

### 3.3 Polyphyllin II inhibits OXPHOS in thyroid cancer cells

**Figure 3:**
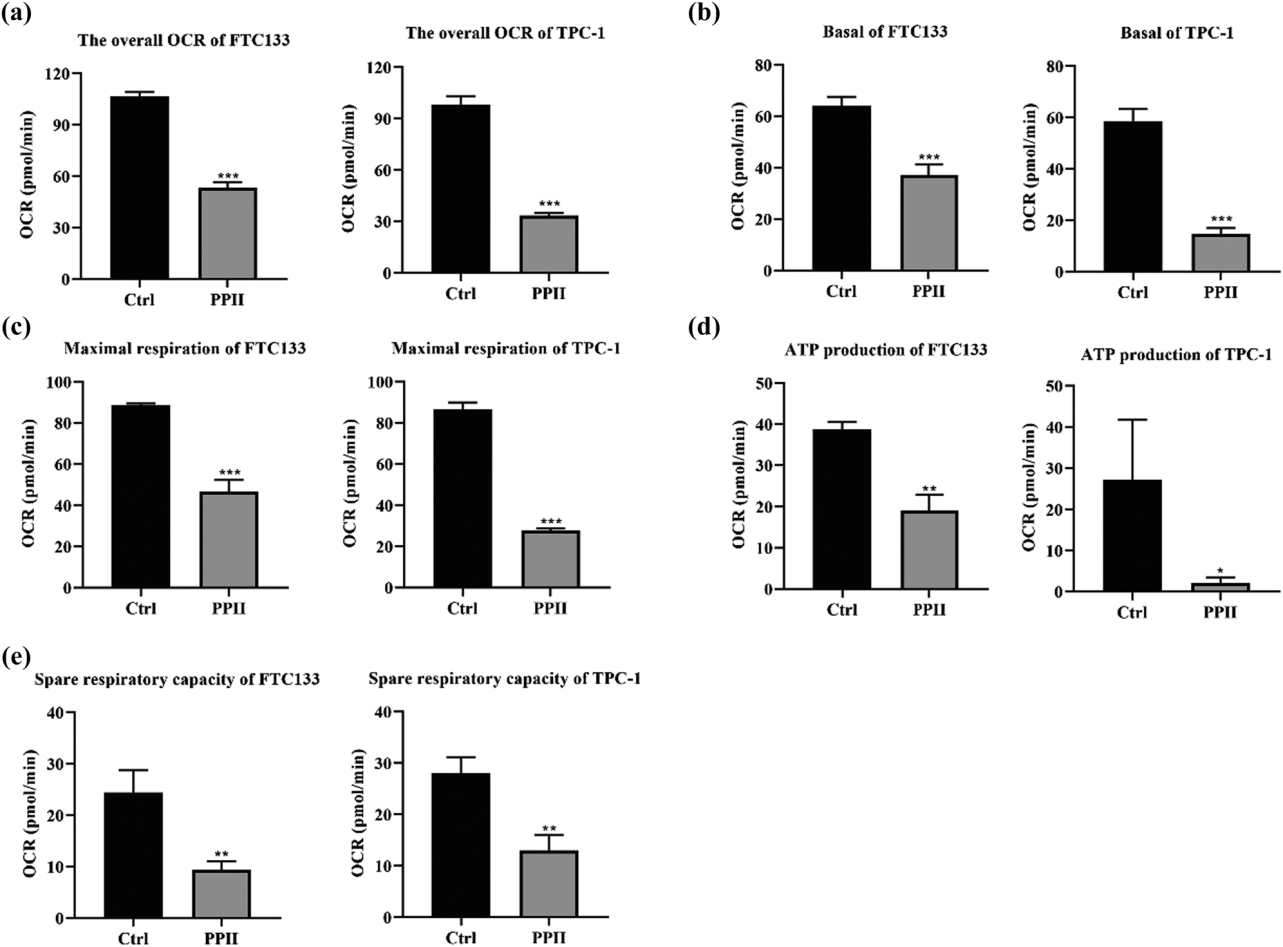
Polyphyllin II inhibits OXPHOS in thyroid cancer cells. (a) OCR assay kit was used to measure overall OCR. (b)–(e) Quantitative data on basal respiration (b), ATP-linked respiration (d), maximal respiration (c), and spare respiratory capacity (e) in FTC133 and TPC-1 cells. Compared with Ctrl, *p < 0.05, **p < 0.01, ***p < 0.001.

To investigate the effect of Polyphyllin II on OXPHOS in thyroid cancer cells, we measured the extracellular OCR. Polyphyllin II significantly reduced the overall OCR in FTC133 and TPC-1 cells (Figure 3a). Statistical analysis showed that Polyphyllin II treatment significantly reduced basal respiration (Figure 3b), maximum respiration (Figure 3c), ATP linked respiration (Figure 3d), and spare respiratory capacity in FTC133 and TPC-1 cells (Figure 3e). From this, it can be seen that Polyphyllin II significantly inhibits the OXPHOS activity in thyroid cancer cells.

### 3.4 Polyphyllin II inhibits glycolysis in thyroid cancer cells

**Figure 4:**
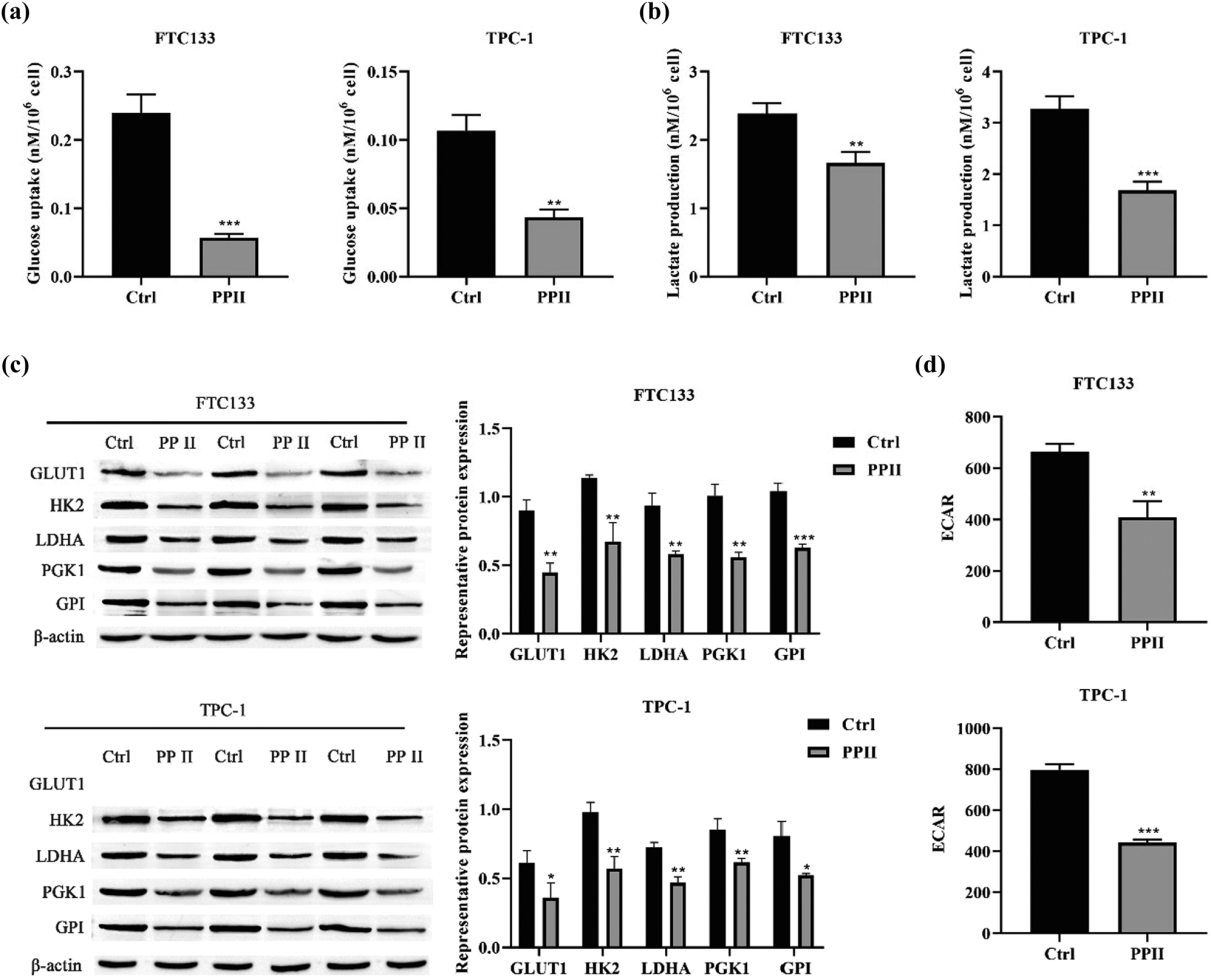
Polyphyllin II inhibits glycolysis in thyroid cancer cells. (a) Glucose detection kit was used to measure cellular glucose uptake. (b) Lactate assay kit was used to quantify lactate production. (c) Western blot was used to detect the expression of glycolytic rate-limiting enzymes. β-actin was used as an internal control. (d) ECAR assay kit was used to measure ECAR. Compared with Ctrl, *p < 0.05, **p < 0.01, ***p < 0.001.

Enhanced aerobic glycolysis, elevated lactate secretion, and increased glucose uptake are characteristics of cellular glycolysis [16]. Therefore, we investigated the effects of Polyphyllin II on glucose uptake, lactate production, glycolytic rate-limiting enzymes, and ECAR in thyroid cancer cells. The results showed that Polyphyllin II significantly reduced glucose consumption (Figure 4a) and lactate production (Figure 4b) in thyroid cancer cells. Western blot was used to detect the expression of glycolytic rate-limiting enzymes, including GLUT1, HK2, LDHA, PGK1, and GPI (Figure 4c). ECAR measurements further demonstrated that treatment with polyphyllin II decreased ECAR in thyroid cancer cells (Figure 4d). Collectively, these findings indicate that polyphyllin II inhibits glycolysis in thyroid cancer cells.

### 3.5 Polyphyllin promotes apoptosis in thyroid cancer cells

**Figure 5:**
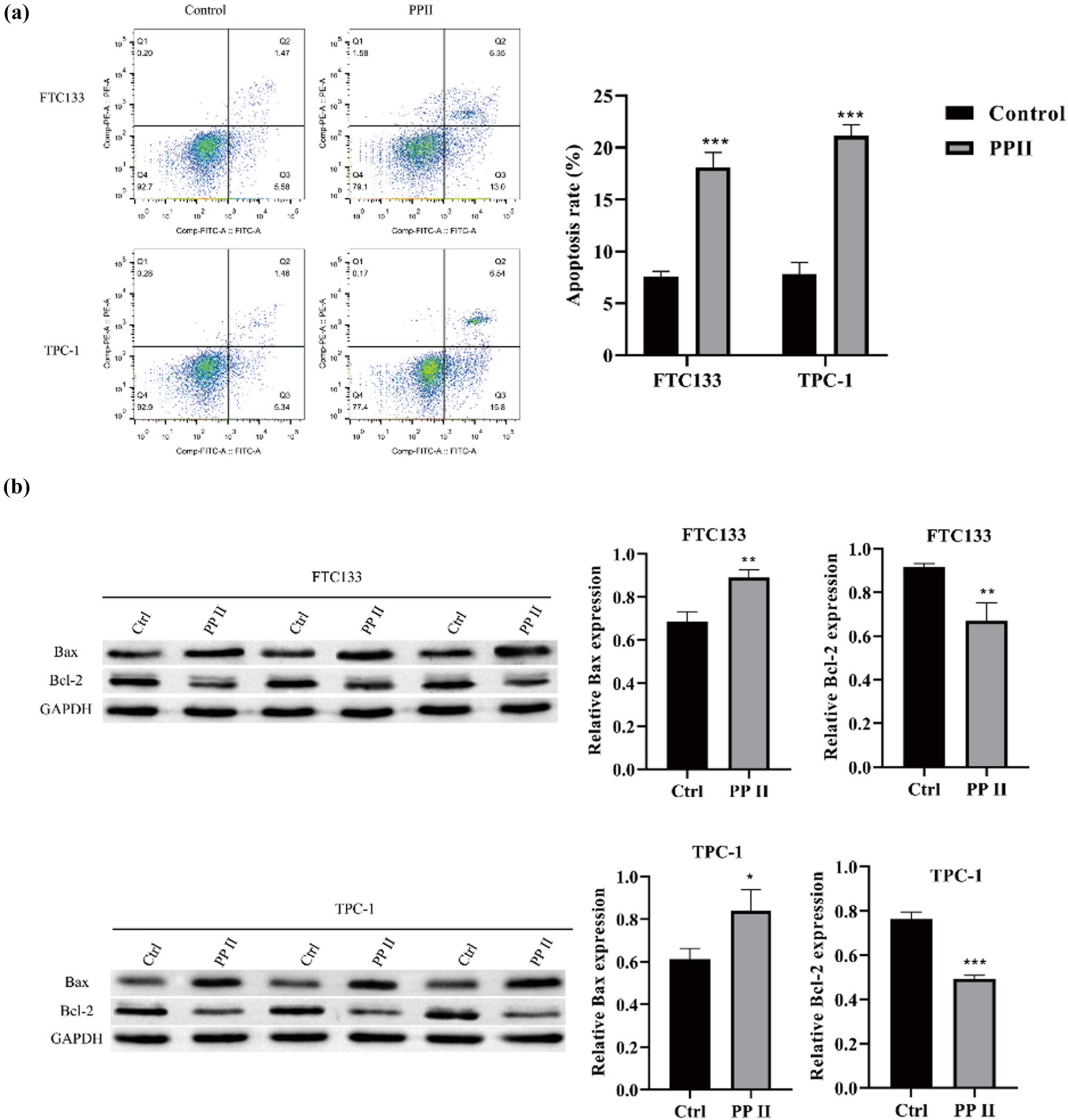
Polyphyllin promotes apoptosis in thyroid cancer cells. (a) Flow cytometry was used to detect cell apoptosis. (b) Western blot was performed to detect the expression of apoptosis-related proteins. GAPDH was used as an internal control. Compared with Ctrl, *p < 0.05, **p < 0.01, ***p < 0.001.

FTC133 and TPC-1 cells were treated with 1 μM Polyphyllin II to investigate its effect on thyroid cancer cell apoptosis. Apoptosis levels were assessed by flow cytometry, and the expression of apoptosis-related proteins was analyzed by western blot in both cell lines. The results showed that Polyphyllin II significantly upregulated apoptosis levels in FTC133 and TPC-1 cells (as shown in Figure 5a and b).

## 4 Discussion

Therapeutic benefits of traditional Chinese medicine in cancer treatment have gained increasing recognition in recent years [17]. Polyphyllin, a saponin extracted from the traditional Chinese medicine *Rhizoma Paridis*, has been identified to show for its broad-spectrum anti-tumor activity [18]. However, the composition of Polyphyllin is complex and it exerts varying degrees of anti-cancer effects in different types of cancer [19]. For example, Lin et al. [20] reported that Polyphyllin VI and VII can induce apoptosis in lung cancer cells both *in vivo* and *in vitro*, with Polyphyllin VII showing a stronger anti-cancer effect than Polyphyllin VI. Xiao et al. [21] reported that Polyphyllin II promotes the apoptosis of ovarian cancer cells, exerting its anti-tumor effect. Han et al. [22] reported that Polyphyllin I suppresses proliferation and promotes apoptosis in gastric cancer cell by inhibiting stat3 phosphorylation. However, there are no reports on whether Rhizoma Paridis saponins have anti-cancer effects on thyroid cancer. In this study, Polyphyllin I, II, III, VI, and VII were selected to evaluate their effects on thyroid cancer. The results showed that all forms of Polyphyllin strongly inhibited FTC133 and TPC-1 thyroid cancer cells, with Polyphyllin II having the most significant effect. Polyphyllin II also inhibited the invasion and migration of thyroid cancer cells. However, high doses of Polyphyllin were cytotoxic to normal human thyroid cells (Nthy-ori 3-1). Zujun et al. discovered that Polyphyllin VII significantly reduces the viability of human non-small cell lung cancer circulating tumor cells (CTC-TJH-01), human lung adenocarcinoma cells (H1975), and human bronchial epithelial-like cells (16HBE) [23]. Similarly, Yang et al. observed mild cytotoxicity of Polyphyllin D mild cytotoxicity in normal liver cells (LO2) after 48 h of treatment [24]. Additionally, Xiang et al. reported that the IC_50_ of Polyphyllin VII for normal gastric epithelial cells (GES-1) was 8.32 ± 1.24 μM [25]. These findings suggest that the cellular effects of Polyphyllin may be closely linked to its underlying mechanisms of action. Studies analyzing the target genes of 12 Polyphyllin components revealed strong associations with key cellular processes, including proliferation, migration, and senescence. Further enrichment analysis indicated that Polyphyllin primarily exerts its effects by inhibiting the phosphoinositide 3-kinase (PI3K)/protein kinase B (Akt) signaling pathway [26].

In thyroid tumors, RAS gene mutations can persistently activate the PI3K/AKT signaling pathway, thereby enhancing glycolytic flux and promoting the membrane localization of glucose transporter protein (GLUT1), which increases glucose uptake capacity. At the same time, the high expression and activity of hexokinase 2 (HK2) play a critical role in the metabolic adaptation of thyroid cancer: by binding to mitochondria, HK2 not only improves metabolic efficiency but also reduces ROS production and prevents apoptosis, significantly promoting tumor cell survival. In addition, studies have shown that AMP-activated protein kinase (AMPK) activity is elevated in papillary thyroid carcinoma cells, which is closely associated with their dependence on glucose metabolism. Meanwhile, pyruvate kinase M2 (PKM2) facilitates a metabolic shift from OXPHOS to aerobic glycolysis (the Warburg effect), meeting the demands of rapidly proliferating tumor cells for nucleotides, amino acids, and lipids, thereby promoting cell proliferation and migration [27]. Molecular docking studies have revealed that PPII can directly bind to AKT targets via hydrogen bonds, thereby interfering with AKT’s function [28]. Furthermore, PPII may regulate the AMPK/mTOR pathway, leading to mitochondrial structural disruption, membrane potential loss, and energy metabolism disorders, and further activating apoptotic signaling pathways [29]. Simultaneously, treatment with PP2 significantly reduced nuclear PKM2 levels, suppressing the transcription of its downstream target genes (such as GLUT1, LDHA, and MYC) and markedly weakening tumor cells’ glycolytic capacity and proliferative ability [30]. However, no studies have yet reported whether Polyphyllin can simultaneously inhibit both OXPHOS and glycolysis in cancer cells. This study is the first to demonstrate that Polyphyllin II suppresses thyroid cancer cell invasion and migration by simultaneously inhibiting both glycolysis and OXPHOS. This finding provides a novel perspective for metabolic targeting therapy in thyroid cancer.

In summary, Polyphyllin I, II, III, VI, and VII were cytotoxic to thyroid cancer cells, with Polyphyllin II being the most effective. Polyphyllin II can inhibit the proliferation, invasion, migration, and promote apoptosis of thyroid cancer cells. Its mechanism may involve simultaneously inhibiting glycolysis and OXPHOS in thyroid cancer cells.

## Figures and Tables

**Figure A1:**
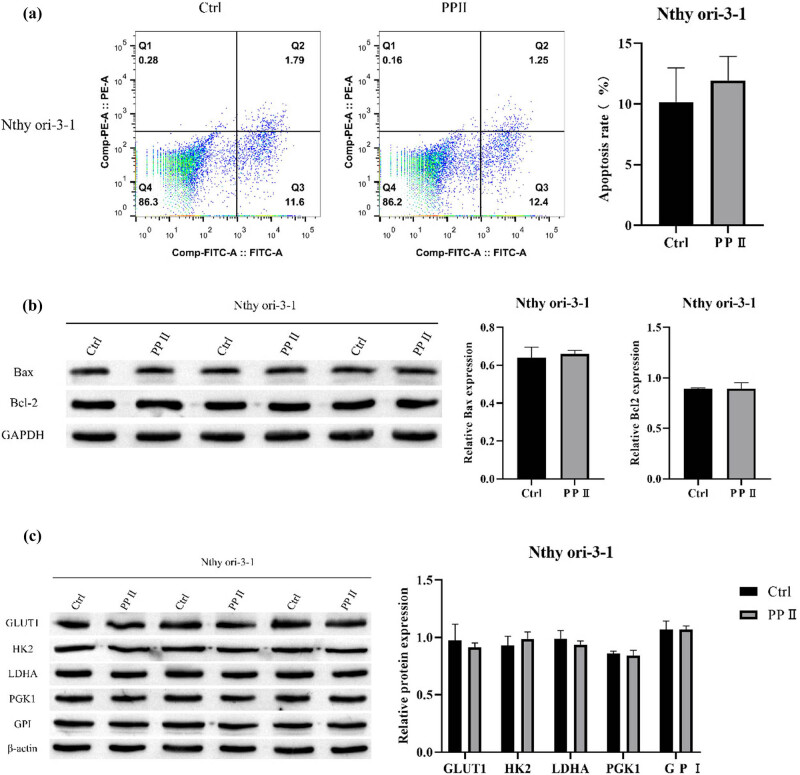
The effect of Polyphyllin Ⅱ on normal thyroid cells. (a) Flow cytometry was used to detect cell apoptosis. (b) Western blot was used to detect the expression of apoptosis-related proteins. GAPDH was used as an internal control. (c) Western blot was used to detect the expression of glycolytic rate-limiting enzymes. β-actin was used as an internal control. Compared with Ctrl, *p < 0.05, **p < 0.01, ***p < 0.001.
